# Methamphetamine and HIV-Tat Protein Synergistically Induce Oxidative Stress and Blood-Brain Barrier Damage via Transient Receptor Potential Melastatin 2 Channel

**DOI:** 10.3389/fphar.2021.619436

**Published:** 2021-03-17

**Authors:** Jian Huang, Ruilin Zhang, Shangwen Wang, Dongxian Zhang, Chi-Kwan Leung, Genmeng Yang, Yuanyuan Li, Liu Liu, Yue Xu, Shucheng Lin, Chan Wang, Xiaofeng Zeng, Juan Li

**Affiliations:** ^1^ NHC Key Laboratory of Drug Addiction Medicine, Kunming Medical University, Kunming, China; ^2^ School of Forensic Medicine, Kunming Medical University, Kunming, China; ^3^ School of Biomedical Sciences, The Chinese University of Hong Kong, Hong Kong, China; ^4^ CUHK-SDU Joint Laboratory of Reproductive Genetics, School of Biomedical Sciences, The Chinese University of Hong Kong, Hong Kong, China; ^5^ School of Basic Medicine, Kunming Medical University, Kunming, China

**Keywords:** transient receptor potential melastatin 2 channel, methamphetamine, HIV-tat protein, blood-brain barrier, oxidative stress

## Abstract

Synergistic impairment of the blood-brain barrier (BBB) induced by methamphetamine (METH) and HIV-Tat protein increases the risk of HIV-associated neurocognitive disorders (HAND) in HIV-positive METH abusers. Studies have shown that oxidative stress plays a vital role in METH- and HIV-Tat-induced damage to the BBB but have not clarified the mechanism. This study uses the human brain microvascular endothelial cell line hCMEC/D3 and tree shrews to investigate whether the transient receptor potential melastatin 2 (TRPM2) channel, a cellular effector of the oxidative stress, might regulate synergistic damage to the BBB caused by METH and HIV-Tat. We showed that METH and HIV-Tat damaged the BBB *in vitro*, producing abnormal cell morphology, increased apoptosis, reduced protein expression of the tight junctions (TJ) including Junctional adhesion molecule A (JAMA) and Occludin, and a junctional associated protein Zonula occludens 1 (ZO1), and increased the flux of sodium fluorescein (NaF) across the hCMEC/D3 cells monolayer. METH and HIV-Tat co-induced the oxidative stress response, reducing catalase (CAT), glutathione peroxidase (GSH-PX), and superoxide dismutase (SOD) activity, as well as increased reactive oxygen species (ROS) and malonaldehyde (MDA) level. Pretreatment with n-acetylcysteine amide (NACA) alleviated the oxidative stress response and BBB damage characterized by improving cell morphology, viability, apoptosis levels, TJ protein expression levels, and NaF flux. METH and HIV-Tat co-induced the activation and high protein expression of the TRPM2 channel, however, early intervention using 8-Bromoadenosine-5′-O-diphosphoribose (8-Br-ADPR), an inhibitor of TPRM2 channel, or TRPM2 gene knockdown attenuated the BBB damage. Oxidative stress inhibition reduced the activation and high protein expression of the TRPM2 channel in the *in vitro* model, which in turn reduced the oxidative stress response. Further, 8-Br-ADPR attenuated the effects of METH and HIV-Tat on the BBB in tree shrews—namely, down-regulated TJ protein expression and increased BBB permeability to Evans blue (EB) and NaF. In summary, the TRPM2 channel can regulate METH- and HIV-Tat-induced oxidative stress and BBB injury, giving the channel potential for developing drug interventions to reduce BBB injury and neuropsychiatric symptoms in HIV-infected METH abusers.

## Introduction

Methamphetamine (METH), a highly addictive synthetic drug characterized by high central excitability and relapse rates, is widely abused worldwide due to the simple synthesis process and low production cost (Huang et al., 2020). Long-term METH abuse damages the central nervous system (CNS), and the resultant neurotoxicity involves multiple mechanisms, including dopaminergic nerve terminal injury, neuronal excitatory toxicity, mitochondrial dysfunction, endoplasmic reticulum stress, neuroinflammation, and oxidative stress response (Mediouni et al., 2015; Northrop and Yamamoto, 2015; Gonçalves et al., 2017; Qie et al., 2017; Yang et al., 2018).

METH use is not uncommon among those infected with HIV, another major public health problem in the world today. Data from the Joint United Nations Programme on HIV/AIDS (UNAIDS) suggests that 37.9 m people were living with HIV at the end of 2018. When HIV-positive patients inject METH intravenously, they increase the risk of spreading HIV by sharing syringes. In the early stage of HIV infection, the virus can enter the CNS and induce HIV-associated neurocognitive disorders (HAND) (Atluri et al., 2015). HAND persistence is influenced by several factors, such as increased life expectancy with antiretroviral therapy, residual levels of the virus in patients’ CNS, and the presence of HIV regulatory proteins such as HIV-Tat in the brain (Mediouni et al., 2015). HIV-Tat encoded by the HIV gene can not only activate HIV transcription and promote HIV replication (Atluri et al., 2015; Mediouni et al., 2015) but also cause neurotoxicity through neuronal excitatory toxicity, mitochondrial dysfunction, endoplasmic reticulum stress, glial cell activation, and oxidative stress response (Ma et al., 2014; Mediouni et al., 2015).

METH and HIV-Tat have a complex interaction. Mediouni et al. (Mediouni et al., 2015) believe that METH abuse can enhance the neurotoxicity of HIV-Tat, and their combined effects can lead to neurotransmitter metabolism disorder, oxidative stress response, and neuroinflammation. Other studies have shown that METH and HIV-Tat can co-induce autophagy and apoptosis of nerve cells (Qi et al., 2011; Li et al., 2018a; Zeng et al., 2018). To become toxic in the CNS, METH, and HIV must first break through the blood-brain barrier (BBB). The BBB is the diffusion barrier between the brain microvascular wall and the brain parenchyma, which is composed of brain microvascular endothelial cells (BMECs), tight junctions (TJs), pericytes, the basement membrane, and the astrocytes. TJs form the basic structure of the BBB, which is composed of three integral membrane proteins—Junctional adhesion molecule (JAM), Occludin, and Claudin, and some cytoplasmic accessory proteins—Zonula occludens (ZO)1, ZO_2_, ZO_3_, and others (Ballabh et al., 2004).

The BBB is a protective and selective permeability barrier that lets water, glucose, amino acids, and some fat-soluble molecules freely penetrate it while restricting neurotoxic substances (Abbott et al., 2006; Campos-Bedolla et al., 2014). However, when METH and HIV-Tat induce neurotoxicity, BBB injury often occurs. Several studies have shown that both METH and HIV-Tat can induce BMEC apoptosis (Ma et al., 2014; Jumnongprakhon et al., 2016; Jumnongprakhon et al., 2017; Qie et al., 2017), destroy the BMEC cytoskeleton (Avraham et al., 2004; Fernandes et al., 2014; Xue et al., 2019), reduce BMEC transepithelial electrical resistance (TEER) (Jumnongprakhon et al., 2016; Patel et al., 2017; Qie et al., 2017), and affect the expression and function of TJ proteins (Xu et al., 2012; Fernandes et al., 2014; Jiang et al., 2017b; Gonçalves et al., 2017; Qie et al., 2017; Xue et al., 2019), thus altering the BBB’s permeability and destroying its structural integrity (Mcrae, 2016; Namyen et al., 2020). Moreover, when a combined exposure of both METH and HIV-Tat, the consequent BMEC damage and abnormal TJ protein expression are more serious (Patel et al., 2017; Li et al., 2018b). METH and HIV-Tat-induced synergistic damage to the BBB increase CNS exposure and the risk of HAND. Previous studies have demonstrated that METH and HIV-Tat can co-induce the oxidative stress response (Zeng et al., 2018), which has been shown to play an important role in BBB injury in rats (Li et al., 2018b). The exact mechanism for this process, however, remains unclear.

The transient receptor potential melastatin 2 (TRPM2) channel, a cation channel that belongs to the transient receptor potential superfamily, has gained much interest in recent years (Belrose and Jackson, 2018; Sita et al., 2018; Wang et al., 2018). It is widely distributed in tissues and cells such as the hippocampus, thalamus, striatum, cerebral cortex, endothelial cells, and glial cells (Turlova et al., 2018). Recent studies have shown that the channel is a cellular effector of oxidative stress and can be activated by many factors, including H_2_O_2_, reactive oxygen species (ROS), and tumor necrosis factor-alpha (TNF-α), therefore, it could mediate oxidative stress response, neuroinflammation, autophagy, and apoptosis (Alawieyah Syed Mortadza et al., 2018; Aminzadeh et al., 2018; Belrose and Jackson, 2018; Miyanohara et al., 2018; Sita et al., 2018; Wang et al., 2020). Besides, inhibition of the TRPM2 channel by 8-Br-cADPR (an inhibitor of the TRPM2 channel) may be a new treatment modality for ischemic acute kidney injury (Eraslan et al., 2019).

Based on the above background research, we hypothesize that the TRPM2 channel can regulate the damaging effects of METH and HIV-Tat on the BBB. Compared with rodents, the tree shrew, a novel model animal, has a more developed brain and is more similar to humans in anatomy, physiology, and genomics (Fan et al., 2013). Recently, the tree shrews have been used as experimental animals in some studies related to METH addiction and toxicity (Li et al., 2018a; Huang et al., 2020). Thus, the human brain microvascular endothelial cell line hCMEC/D3 and tree shrews are used as the research objects for *in vivo* and *in vitro* analyses of how METH and HIV-Tat co-induce oxidative stress injury to damage the BBB, and to identify the TRPM2 channel’s function and mechanism in this process. The results present a novel theory of the BBB injury induced by METH and HIV-Tat and provide a new scientific basis for developing effective drug intervention targets for HIV-positive METH abusers.

## Materials and Methods

### Materials

METH was purchased from the National Institutes for Food and Drug Control (Cat#: 171212-200603, Beijing, China). Recombinant HIV-1 Tat Clade-B was purchased from Prospec (Tat, Cat#: HIV-129, Rehovot, Israel). N-acetylcysteine amide (NACA, Cat#: A0737), 8-Bromo-cyclic adenosine diphosphate ribose (8-Br-cADPR, Cat#: B5416), Evans Blue (EB, Cat#: E2129), and sodium fluorescein (NaF, Cat#: F6377) were purchased from Sigma-Aldrich (Missouri, United States). DeadEnd™ Fluorometric TUNEL System was purchased from Progema (Cat#: G3250, Fitchburg, United States). The antibodies used were JAMA (Cat#: ab180821, 1:1,000, Abcam, United Kingdom), Occludin (Cat#: ab167161, 1:5,000, Abcam, United Kingdom), ZO1 (Cat#: ab216880, 1:1,000, Abcam, United Kingdom), TRPM2 (Cat#: ab11168, 1:1,000, Abcam, United Kingdom), β-Actin (Cat#: 21,338, 1:1,000, Signalway Antibody, United States), and secondary antibody (Cat#: L3012, 1:5,000, Signalway Antibody, United States).

### Animal Experiments

Male tree shrews (120 to 160 g, 1-year-old) were supplied by the Center of Tree Shrew Germplasm Resources, the Institute of Medical Biology, the Chinese Academy of Medical Science, and Peking Union Medical College (Kunming, China). They were housed in a standard 12 h:12 h light/dark cycle at a room temperature of 23 ± 2°C, with access to food and water *ad libitum*, and give humanitarian care according to the 3R principle used in laboratory animals. All experiments were approved by the Institutional Ethics Committee of Kunming Medical University and were performed according to ethical standards described in the NIH guidelines. The tree shrews were randomly divided into six groups: 1) control (C) group; 2) 8-Br-cADPR (8-Br) group; 3) METH (M) group; 4) HIV-Tat (T) group; 5) METH + HIV-Tat (M + T) group; 6) 8-Br-cADPR + METH + HIV-Tat (8-Br + M + T) group. The animals in groups 1) and 2) were intraperitoneally injected with either saline (0.2 ml) or 8-Br-cADPR (40 μg/kg); 3) to 5) were injected with METH (8 mg/kg, intraperitoneal injection) and/or HIV-Tat (100 ng, tail intravenous injection); 6) were injected with 8-Br-cADPR (40 μg/kg, intraperitoneal injection) 0.5 h before exposure to METH and HIV-Tat. All the animals were treated for 10 consecutive days per the above requirements and euthanized 24 h after the final treatment. Their brains were then harvested and snap-frozen in liquid nitrogen for further analysis.

### Evaluation of BBB Permeability in Brain Tissues

Six animals in each group were treated with EB (2%, 2 ml/kg, n = 3) or NaF (2%, 2 ml/kg, n = 3) by tail intravenous injection 1 h before the end of the experiment. Approximately 0.5 h later, the animals were euthanized with sodium pentobarbital (10%, 3 ml/kg) by intraperitoneal injection and perfused with 300 ml of heparinized saline (0.9% sodium chloride and 20 U/ml sodium heparin). The brains injected with EB were harvested and dipped in dimethyl sulfoxide (1 ml/100 mg brain tissue) for 24 h at 60°C. After centrifugation at 1000 g for 5 min, optical densities (ODs) of the supernatants were measured by spectrophotometer at 550 nm excitation wavelength and 620 nm emission wavelength to evaluate the EB content. Another three brains injected with NaF were then harvested and dipped in 5% trichloroacetic acid (1 ml/100 mg brain tissue). Following centrifugation at 12000 g for 5 min, the supernatants were taken and 5 mol/L NaOH (1:0.8) matched into the samples. ODs of the samples were then measured by spectrophotometer at 450 nm excitation wavelength and 525 nm emission wavelength to evaluate the NaF content.

### Cell Cultures and Treatments

The human brain microvascular endothelial cell line hCMEC/D3 cells were purchased from the National Infrastructure of Cell Line Resource (Beijing, China). The cells were derived from human brain microvascular endothelium and shared characteristics with the BBB, including the expression of TJ proteins (Weksler et al., 2013). The cells were cultured in DMEM/high glucose (Hyclone, United States) supplemented with 10% FBS (Gibco, United States), 1% penicillin/streptomycin (Gibco, United States), and 1 ng/ml human basic fibroblast growth factor (Sigma-Aldrich, United States) in 5% CO_2_ at 37°C. According to the experimental requirements, the cells were plated in 6-well plates, 24-well plates, or 96-well plates, respectively, and treated with different drugs for different durations when the cells reached appropriate confluence. A non-serum medium was used for drug treatments.

### Lentiviral Transfection

shTRPM2 lentivirus (LV-shTRPM2) and shNC lentivirus (LV-shNC) were obtained from GenePharma Co., Ltd (Shanghai, China). The sequences were as follows: shTRPM2: 5′-GCA​ATA​AGG​TTG​ACG​CCA​TGG-3′; shNC (refers to an empty vector): 5′-TTC​TCC​GAA​CGT​GTC​ACG​T-3′. The titer of the virus was 5 × 10^8^ TU/ml. According to the experimental requirements, the cells were plated in 6-well plates, 24-well plates, or 96-well plates, and transfection with LV-shTRPM2 or LV-shNC (1:50, containing 5 μg/ml polybrene) was conducted when the cells reached 40% confluence. The medium was replaced 24 h after infection. The cells were further cultured for 24 h to 48 h, then used for subsequent experiments.

### Real-Time qPCR

Total RNA was extracted from the cells infected by LV-shTRPM2 or LV-shNC using the TRIzol reagent (Invitrogen, United States). According to the manufacturer’s instructions, the total RNA was synthesized into cDNA using the First Strand cDNA Synthesis Kit (Thermo Scientific, United States), and the real-time qPCR reaction was performed using the FastStart Universal SYBR Green Master (ROX) kit (Roche, Switzerland) in a real-time qPCR system (ABI 7300, United States). The total reaction volume was 20 μL. The relative expression of TRPM2 mRNA was calculated using the 2^−△△CT^ method. Each experiment was performed in triplicate wells and replicated.

The primers were designed and synthesized by Sangon Biotech Co., Ltd (Shanghai, China), and they were compatible with the Minimum Information for Publication of Quantitative Real-Time PCR Experiments (MIQE) guidelines. The primer sequences were as follows:

TRPM2: Sense: 5′-TTC​GTG​GAT​TCC​TGA​AAA​CAT​CA-3′; Antisense: 5′-CCA​GCA​TCA​GAC​AGT​TTG​GAA​C-3′.

GAPDH: Sense: 5′-GAG​CGA​GAT​CCC​TCC​AAA​AT-3′; Antisense: 5′-GCT​GTT​GTC​ATA​CTT​CTC​AT-3′.

### Analysis of Cell Viability

The viability of hCMEC/D3 cells was quantified using the CCK8 kit (Beyotime, Shanghai, China). The cells were plated in 96-well plates and cultured in 5% CO_2_ at 37°C. Following exposure to different drugs for different durations, the medium in each well was discarded and replaced with 90 μL of serum-free medium and 10 μL of CCK8 reagent. After being incubated at 37°C for 2 h, the ODs were determined at 450 nm using a universal microplate reader (BioTek, United States). Each experiment was conducted in triplicate wells and replicated.

### Assessment of Apoptosis

The cells were plated in 24-well plates and cultured in 5% CO2 at 37°C. Following exposure to different drugs for different durations, the cells were fixed in a 4% methanol-free formaldehyde solution and permeabilized with 0.2% Triton® X-100 solution. Then, the cells were incubated with the TUNEL solution (Progma, United States) at 37°C for 1 h. After DAPI (Cat#: H-1200, Vectorlab, United States) were added to each well to stain nuclei, the fluorescence signals were observed by an inverted fluorescence microscope (Nikon TE2000U, Japan). The image capture area was randomly selected by a researcher who was blinded to the experimental conditions. Each experiment was conducted in triplicate wells and replicated, and three images were captured from each well. The apoptosis level was determined by the number of TUNEL-positive cells expressed as a percentage of the total cell number (analyzed by Image J software).

### Measurement of ROS

Intracellular ROS levels were measured using commercial kits (Beyotime, Shanghai, China) according to the manufacturer’s instructions. The cells were seeded briefly in 24-well plates and cultured in 5% CO_2_ at 37°C. Following exposure to different drugs for different durations, 10 μM DCFH-DA via the non-serum medium was added to each well and incubated at 37°C for 20 min. The cells were subsequently washed three times with PBS. DCF fluorescence was measured at 488 nm excitation wavelength and 520 nm emission wavelength using an inverted fluorescence microscope (Nikon TE2000U, Japan). The image capture area was randomly selected by a researcher who was blinded to the experimental conditions. Each experiment was conducted in triplicate wells and replicated, and three images were captured from each well. The fluorescence intensity was analyzed by Image J software.

### Measurement of Antioxidant Enzyme Activity and Malonaldehyde Level

Catalase (CAT), glutathione peroxidase (GSH-PX), and superoxide dismutase (SOD) activity and the malonaldehyde (MDA) level were measured using commercial kits (Nanjing Jiancheng Bioengineering Institute, Nanjing, China) according to the manufacturer instructions. The cells were seeded briefly in 6-well plates and cultured in 5% CO_2_ at 37°C.

The decomposition of H_2_O_2_ by CAT can be stopped quickly by ammonium molybdate. The remaining H_2_O_2_ reacts with ammonium molybdate to form a light-yellow complex, and its absorbance measured at 405 nm can be used to calculate the CAT activity. GSH-PX can catalyze the reaction of H_2_O_2_ with GSH to generate H_2_O and oxidized glutathione (GSSG). The consumption of GSH can reflect the activity of GSH-PX in this enzymatic reaction. GSH reacts with dithiodinitrobenzoic acid to produce a 5-thiodinitrobenzoic acid anion, which presents a relatively stable yellow color. Measuring its absorbance at 412 nm can be used to calculate the GSH content and indirectly calculate GSH-PX activity. The superoxide anion radical (O_2-_·) produced by the xanthine/xanthine oxidase reaction can oxidize hydroxylamine to form nitrite, which will appear purple-red under the action of the color reagent. SOD has a specific inhibitory effect on O_2-_·, so it can weaken the color reaction. Measuring its absorbance at 550 nm can be used to calculate the SOD activity. MDA can be condensed with thiobarbituric acid to form a red product, and its absorbance measured at 530 nm can be used to calculate the MDA level. Each experiment was conducted in triplicate wells and replicated.

### Determination of Ca^2+^ Concentration

The concentration of Ca^2+^ in the hCMEC/D3 cells was determined via fluorescent probe Fura-2 AM (Invitrogen, USA). The cells were seeded briefly in 24-well plates and cultured in 5% CO_2_ at 37°C. Following exposure to different drugs for different durations, the cells were loaded with 4 μM Fura-2 AM in Hank’s balanced salt solution (HBSS) and incubated at 37°C for 50 min. The cells were then washed three times with HBSS, and further incubated at 37°C for 30 min. Ca^2+^ fluorescence was measured at 340/380 nm excitation wavelength and 510 nm emission wavelength using an inverted fluorescence microscope (Nikon TE2000U, Japan). The image capture area was randomly selected by a researcher who was blinded to the experimental conditions. Each experiment was conducted in triplicate wells and replicated, and three images were captured from each well. The fluorescence intensity was analyzed by Image J software.

### Evaluation of the hCMEC/D3 Cells Permeability

The flux of NaF across the hCMEC/D3 cells was used to determine the permeability of brain endothelial monolayers. The cells were seeded in the upper inserts (Millipore, United States) of the Transwell system and cultured until the formation of a tight monolayer. Following exposure to different drugs for different durations, the medium in the Transwell system was replaced with HBSS, and 10 μg/ml NaF was added to the upper insert. After the cells were incubated at 37°C for 1 h, 100 μL medium was collected from the acceptor chamber. ODs of the samples were measured at 485 nm excitation wavelength and 535 nm emission wavelength by fluorescence multi-plate reader (BioTek, United States) to evaluate the concentrations of NaF from the top to bottom chamber. Each experiment was conducted in triplicate wells and replicated.

### Western Blot Assay in Cells and Brain Tissues

The cells or brain tissues were washed twice with cold PBS, then homogenized in a protein extraction buffer (Beyotime, Shanghai, China) containing protease and phosphatase inhibitors and centrifuged at 14,000 g at 4°C for 15 min. The supernatant was collected, and the protein concentrations were measured using the Bradford Protein Assay kit (Beyotime, Shanghai, China). After the protein sample loading buffer was added, the samples were boiled at 99°C for 10 min. The samples were then separated by 8% SDS-PAGE and transferred to 0.45 µm polyvinylidene difluoride membranes (Millipore, Billerica, MA, United States). The membranes were blocked in 5% non-fat dry milk (diluted in the tris-buffered saline with 0.1% Tween 20 (TBST)) at room temperature for 1 h, then incubated in appropriate primary antibodies (diluted with 5% defatted milk) overnight at 4°C. Next, the membranes were washed three times for 10 min each with TBST and incubated with the secondary antibody (diluted with 5% defatted milk) at room temperature for 1 h. Finally, the membranes were detected using an enhanced chemiluminescent Plus Detection kit (Millipore, United States) and visualized using a Bio-Rad Imaging system (Bio-Rad, United States). This experiment was repeated in triplicate, and representative Western blot images were presented.

### Data Analysis

Statistical analyses were performed using SPSS 21 (IBM SPSS, Chicago, United States) and GraphPad Prism 8 (GraphPad Software, United States). All data were represented as the mean ± SD. One-way ANOVA tests (analyzed post-hoc using the LSD test) and Student’s t-tests were performed. *p* < 0.05 was considered statistically significant. The Bliss Independent model (Foucquier and Guedj, 2015) was used to calculate whether the changes in related indicators caused by METH and HIV-Tat had a synergistic effect. The calculation formula was as follows: (Effect_METH_ + Effect_Tat_ − Effect_METH_ × Effect_Tat_)/Effect_METH+Tat_. Effect_METH/Tat/METH+Tat_ had to be normalized to ensure the value between 0 and one using the Max–Min formula: Zi = Xi−Min/Max−Min. According to the calculation formula, the effect value < 1 meant synergistic action, = 1 meant additive action, and >1 meant antagonistic action.

## Results

### METH and HIV-Tat Induce Synergistic Injury to the BBB *in vitro*

**FIGURE 1:**
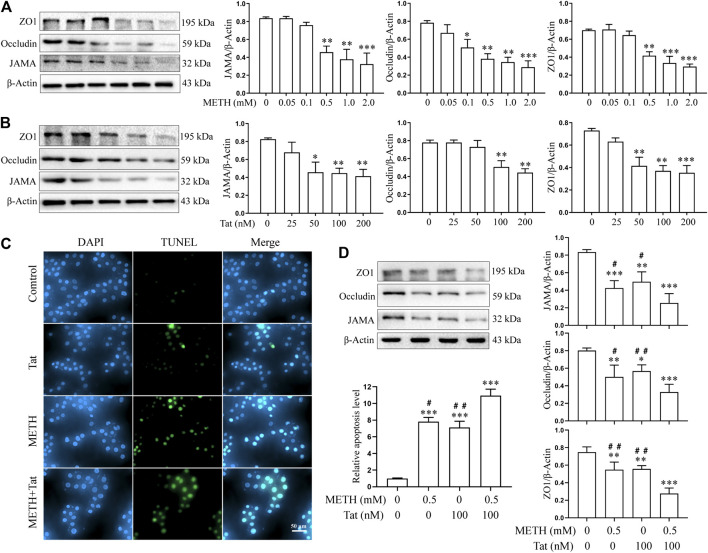
METH and HIV-Tat synergistically damage the BBB model *in vitro*. **(A, B)** Western blot was performed to determine JAMA, Occludin, and ZO1 expression levels in the hCMEC/D3 cells treated with a gradient concentration of METH or HIV-Tat for 24 h. After the cells were treated with 0.5 mM of METH and/or 100 nM of HIV-Tat for 24 h, **(C)** apoptosis was assessed by TUNEL assay (Scale bar: 50 µm), apoptosis level was determined by the number of TUNEL-positive cells expressed as a percentage of the total cell number (analyzed by Image J software); **(D)** western blot was performed to determine JAMA, Occludin, and ZO1 expression levels. Compared with the control, *p < 0.05, **p < 0.01, ***p < 0.001; compared with the METH + HIV-Tat, ^#^ *p* < 0.05, ^##^ *p* < 0.01, n = 3.

The hCMEC/D3 cells were treated with METH (0.05 to 2.0 mM) or HIV-Tat (25 to 200 nM) for 24 h. As the drug concentrations increased, the expression levels of JAMA, Occludin, and ZO1 all showed a downward trend (Figures 1A,B). Compared with the control group, 0.5 mM METH and 100 nM HIV-Tat induced significant decreases in TJ protein expression levels (Figures 1A,B). This regimen was selected for subsequent cell experiments. Through an inverted microscope, the cells in the control group (C) were observed to have a full and transparent cell body, good morphology and refractive index, a smooth cell membrane, and a clear boundary. In the group METH (M), HIV-Tat (T), and METH + HIV-Tat (M + T), many cells shrank and became round. Some cells showed vacuole-like structures in their cytoplasm, and a few cells even floated off the wall and died (Supplementary Figure S1A). Compared with group C, the apoptosis levels in groups M, T, and M + T were significantly higher (Figure 1C), TJ protein expression levels significantly lower (Figure 1D), and the flux of NaF across the hCMEC/D3 cells were significantly higher (Supplementary Figure S2B). These indicators of the cells in group M + T changed more obvious compared to those in group M or T. The effect values were 0.879 (apoptosis), 0.879 (JAMA), 0.814 (Occludin), 0.657 (ZO1), and 0.821 (NaF), showing a synergistic effect (Figures 1C,D and Supplementary Figure S2B). These results demonstrate that METH and HIV-Tat jointly damage the BBB.

### METH and HIV-Tat Co-induce Oxidative Stress to Damage the BBB *in vitro*

**FIGURE 2:**
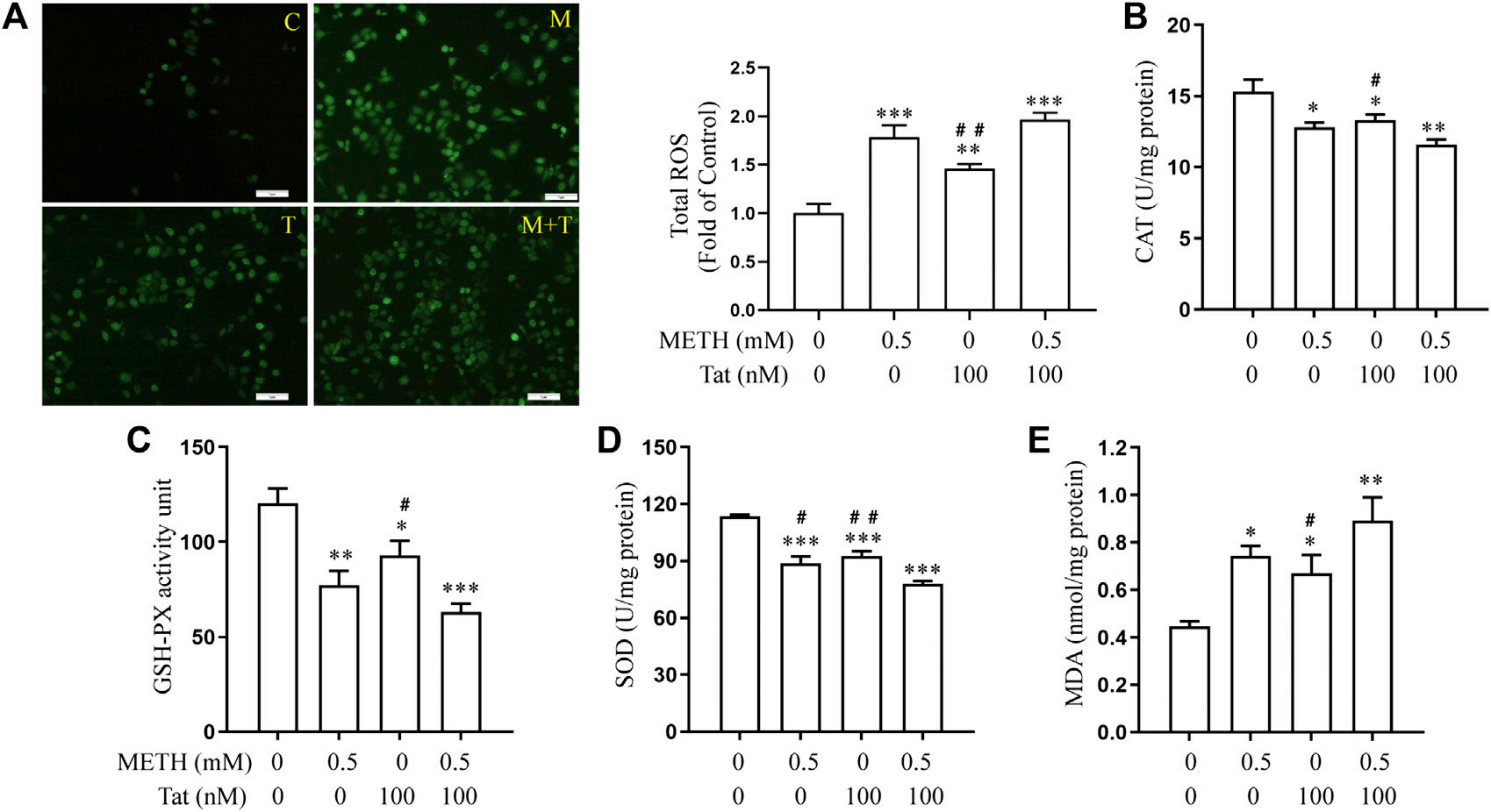
METH and HIV-Tat synergistically induce oxidative stress responses. After the hCMEC/D3 cells were treated with 0.5 mM of METH and/or 100 nM of HIV-Tat for 24 h, **(A)** the ROS fluorescence was observed by an inverted fluorescence microscope (Scale bar: 1 µm), and the fluorescence intensity was analyzed by Image J software; **(B–E)** the activity of CAT, GSH-PX, and SOD and the level of MDA were measured using commercial kits. Compared with the control, *p < 0.05, **p < 0.01, ***p < 0.001; compared with the METH + HIV-Tat, ^#^ *p* < 0.05, ^##^ *p* < 0.01, n = 3. C=Control, M = 0.5 mM of METH, T = 100 nM of HIV-Tat, and M + T = 0.5 mM of METH+100 nM of HIV-Tat.

**FIGURE 3:**
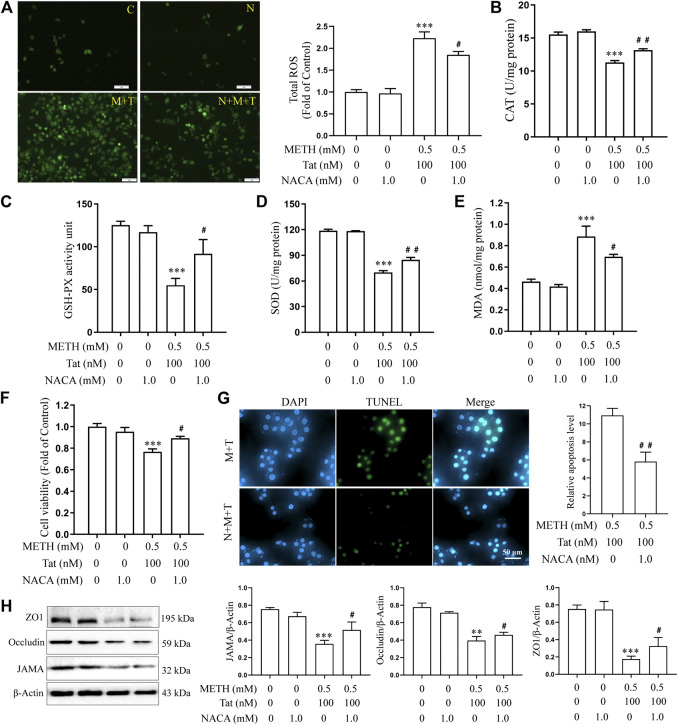
METH and HIV-Tat synergistically induce oxidative stress that damages the BBB model *in vitro*. The hCMEC/D3 cells were treated with or without 1.0 mM of NACA for 1 h before 0.5 mM of METH and 100 nM of HIV-Tat for 24 h. **(A)** ROS fluorescence was observed by an inverted fluorescence microscope (Scale bar: 1 µm), and the fluorescence intensity was analyzed by Image J software; **(B–E)** the activity of CAT, GSH-PX, and SOD and the MDA level were measured using commercial kits; **(F)** the cell viability was measured using the CCK8 kit; **(G)** apoptosis was assessed via TUNEL assay (Scale bar: 50 µm), apoptosis level was determined by the number of TUNEL-positive cells expressed as a percentage of the total cell number (analyzed by Image J software); **(H)** western blot was performed to determine JAMA, Occludin, and ZO1 expression levels. Compared with the control, **p < 0.01, ***p < 0.001; compared with the METH + HIV-Tat, ^#^ *p* < 0.05, ^##^ *p* < 0.01, n = 3. C=Control, N = 1.0 mM of NACA, M + T = 0.5 mM of METH+100 nM of HIV-Tat, and N + M + T = 1.0 mM of NACA+0.5 mM of METH+100 nM of HIV-Tat.

Previous studies have shown that METH- and HIV-Tat-induced injury to the BBB in SD rats is accompanied by a severe oxidative stress response (Li et al., 2018b). In this study, we found that METH and/or HIV-Tat decreased the CAT (Figure 2B), GSH-PX (Figure 2C), and SOD (Figure 2D) activities of the cells, while increased the ROS (Figure 2A) and MDA (Figure 2E) levels. The changes in these indicators of the cells were more obvious in the group M + T compared with group M or T, where the effect values were 0.845 (CAT), 0.872 (GSH-PX), 0.876 (SOD), 0.903 (ROS), and 0.834 (MDA), indicating a synergic effect (Figures 2A–E). Then, we used the antioxidant NACA (1.0 mM, treat the cells for 1 h in advance) (Supplementary Figure S1B) to investigate the combined damaging effects of oxidative stress, HIV-Tat, and METH on the BBB. The cells were divided into four groups: control (C), NACA (N), METH + HIV-Tat (M + T), and NACA + METH + HIV-Tat (N + M + T). The NACA intervention improved the severe oxidative stress that had been synergistically induced by METH and HIV-Tat (Figures 3A–E). Furthermore, compared with the group M + T, the cell morphology (Supplementary Figure S1C), viability (Figure 3F), apoptosis levels (Figure 3G), TJ protein expression levels (Figure 3H), and NaF flux (Supplementary Figure S2B) all improved in the group N + M + T. These results demonstrate that METH and HIV-Tat co-induce oxidative stress to damage the BBB.

### TRPM2 Channel Regulates METH- and HIV-Tat-Induced Synergistic Injury to the BBB *in vitro*

**FIGURE 4:**
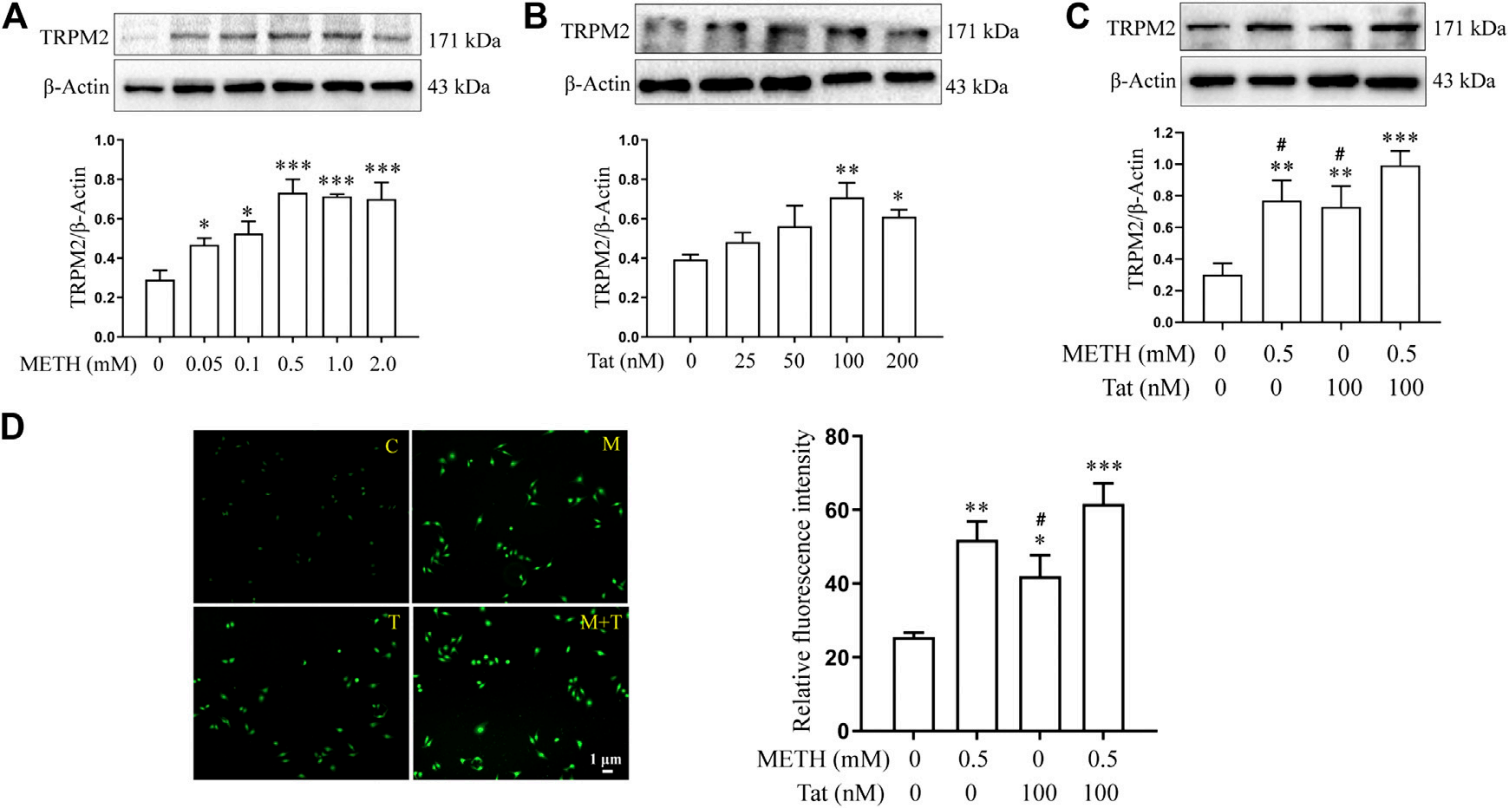
METH and HIV-Tat synergistically activate the TRPM2 channel. **(A,B)** Western blot was performed to determine TRPM2 expression level in the hCMEC/D3 cells treated with a gradient concentration of METH or HIV-Tat for 24 h. After the cells were treated with 0.5 mM of METH and/or 100 nM of HIV-Tat for 24 h, **(C)** western blot was performed to determine the TRPM2 expression level; **(D)** the Ca^2+^ fluorescence was observed using an inverted fluorescence microscope (Scale bar: 1 µm), and the fluorescence intensity was analyzed using Image J software. Compared with the control, *p < 0.05, **p < 0.01, ***p < 0.001; compared with the METH + HIV-Tat, ^#^ *p* < 0.05, n = 3. C=Control, M = 0.5 mM of METH, T = 100 nM of HIV-Tat, and M + T = 0.5 mM of METH+100 nM of HIV-Tat. The Ca^2+^ fluorescence shown in **(D)** was detected after the cells were treated with 0.5 mM of METH and/or 100 nM of HIV-Tat for 0.5 h.

We then investigated whether the TRPM2 channel could regulate METH- and HIV-Tat-induced synergistic injury to the BBB *in vitro*. The hCMEC/D3 cells were transfected with LV-shTRPM2 or LV-shNC for 48 h, then western blot was performed to validate TRPM2 protein. We found that the band sized at 171 kDa was significantly decreased in group LV-shTRPM2 compared with the group LV-NC (Supplementary Figure S3). Thus, we confirmed that this antibody is indeed targeting the TRPM2 protein and the band sized at 171 kDa is the TRPM2 protein band. The TRPM2 protein expression level increased by different degrees after the hCMEC/D3 cells were treated with METH (0.05 to 2.0 mM) or HIV-Tat (25 to 200 nM) for 24 h (Figures 4A,B). Compared to the cells treated with 0.5 mM METH or 100 nM HIV-Tat alone for 24 h, cells, where the two were combined, showed a more significant increase in TRPM2 protein expression, indicating a synergic effect (the effect value was 0.878) (Figure 4C). Furthermore, the intensity of Ca^2+^ fluorescence increased significantly in groups M, T, and M + T (METH and/or HIV-Tat exposure for 0.5 h) compared with group C. Compared with groups M or T, this phenomenon in group M + T was more obvious, with a synergistic effect (the effect value was 0.854) (Figure 4D). These results indicate that METH and/or HIV-Tat activate the TRPM2 channel.

**FIGURE 5:**
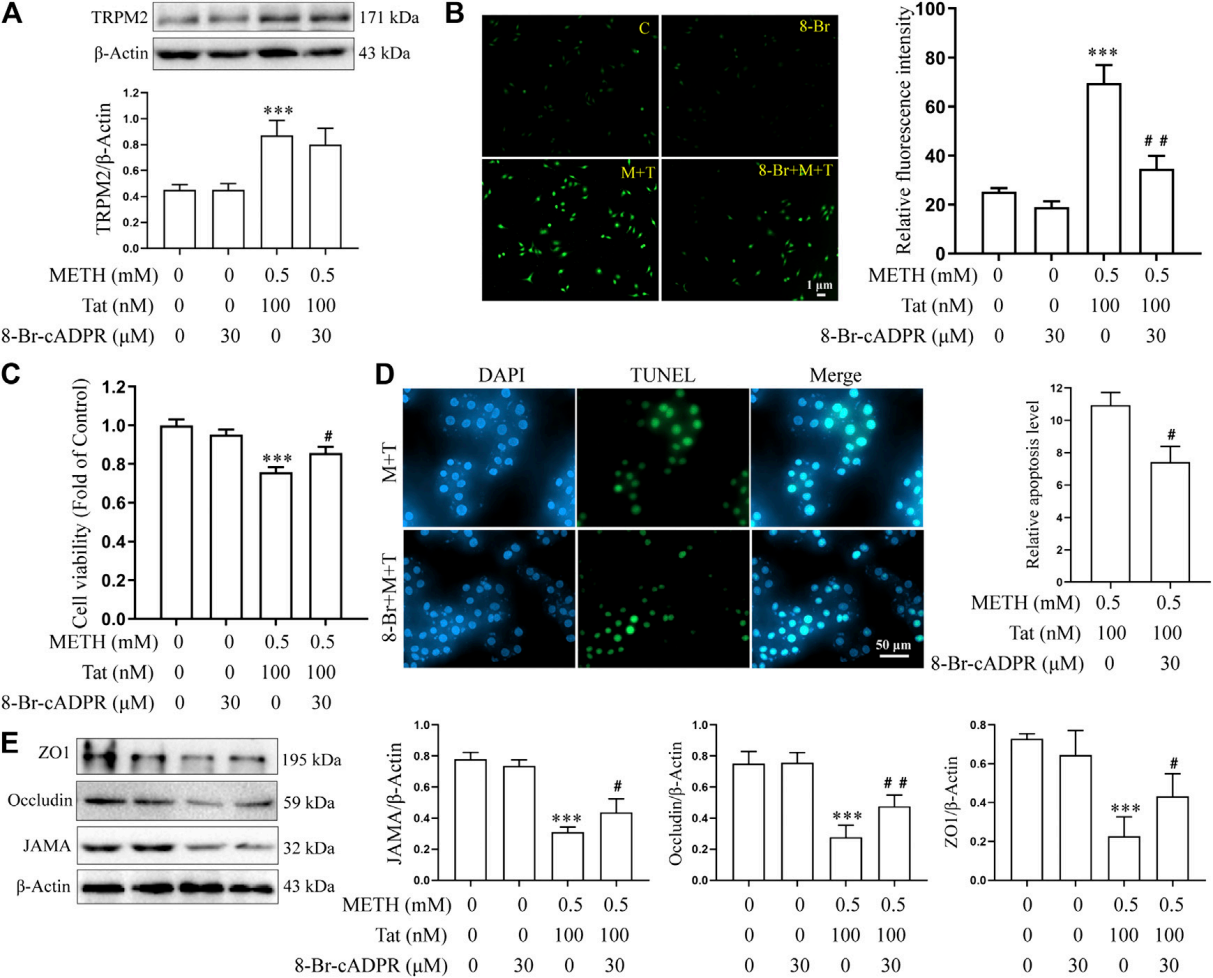
8-Br-cADPR attenuates damage to the BBB induced synergistically by METH and HIV-Tat *in vitro*. The hCMEC/D3 cells were treated with or without 30 μM of 8-Br-cADPR for 0.5 h before 0.5 mM of METH and 100 nM of HIV-Tat for 24 h. **(A)** Western blot was performed to determine the TRPM2 expression level; **(B)** the Ca^2+^ fluorescence was observed via an inverted fluorescence microscope (Scale bar: 1 µm); fluorescence intensity was analyzed using Image J software; **(C)** cell viability was measured using the CCK8 kit; **(D)** apoptosis was assessed via TUNEL assay (Scale bar: 50 µm), apoptosis level was determined by the number of TUNEL-positive cells expressed as a percentage of the total cell number (analyzed by Image J software); **(E)** western blot was performed to determine JAMA, Occludin, and ZO1 expression levels. Compared with the control, ***p < 0.001; compared with the METH + HIV-Tat, ^#^ *p* < 0.05, ^##^ *p* < 0.01, n = 3. C=Control, 8-Br = 30 μM of 8-Br-cADPR, M + T = 0.5 mM of METH+100 nM of HIV-Tat, and 8-Br + M + T = 30 μM of 8-Br-cADPR+0.5 mM of METH+100 nM of HIV-Tat. The Ca^2+^ fluorescence shown in **(B)** was detected after the cells were treated with or without 30 μM of 8-Br-cADPR for 0.5 h before 0.5 mM of METH and 100 nM of HIV-Tat for 0.5 h.

8-BR-CADPR, an inhibitor of the TRPM2 channel (Eraslan et al., 2019; Alves-Lopes et al., 2020), was used to determine the role of the TRPM2 channel in METH- and HIV-Tat-induced co-injury to the BBB *in vitro*. The cells were divided into control (C), 8-BR-CADPR (8-BR), METH + HIV-Tat (M + T), and 8-BR-cadPR + METH + HIV-Tat (8-BR + M + T) groups. 30 μM 8-BR-CADPR was used to treat the cells for 0.5 h in advance according to the CCK8 results (Supplementary Figure S1D). We found that the 8-BR-CADPR intervention did not reduce the high TRPM2 protein expression caused by METH and HIV-Tat (Figure 5A). It was able, however, to reduce intracellular Ca^2+^ fluorescence intensity (METH and HIV-Tat exposure for 0.5 h) (Figure 5B), indicating that the TRPM2 channel had been inhibited. This inhibition improved the abnormal cell morphology (Supplementary Figure S1E), viability (Figure 5C), apoptosis levels (Figure 5D), TJ protein expression levels (Figure 5E), and NaF flux (Supplementary Figure S2B) caused by METH and HIV-Tat.

**FIGURE 6:**
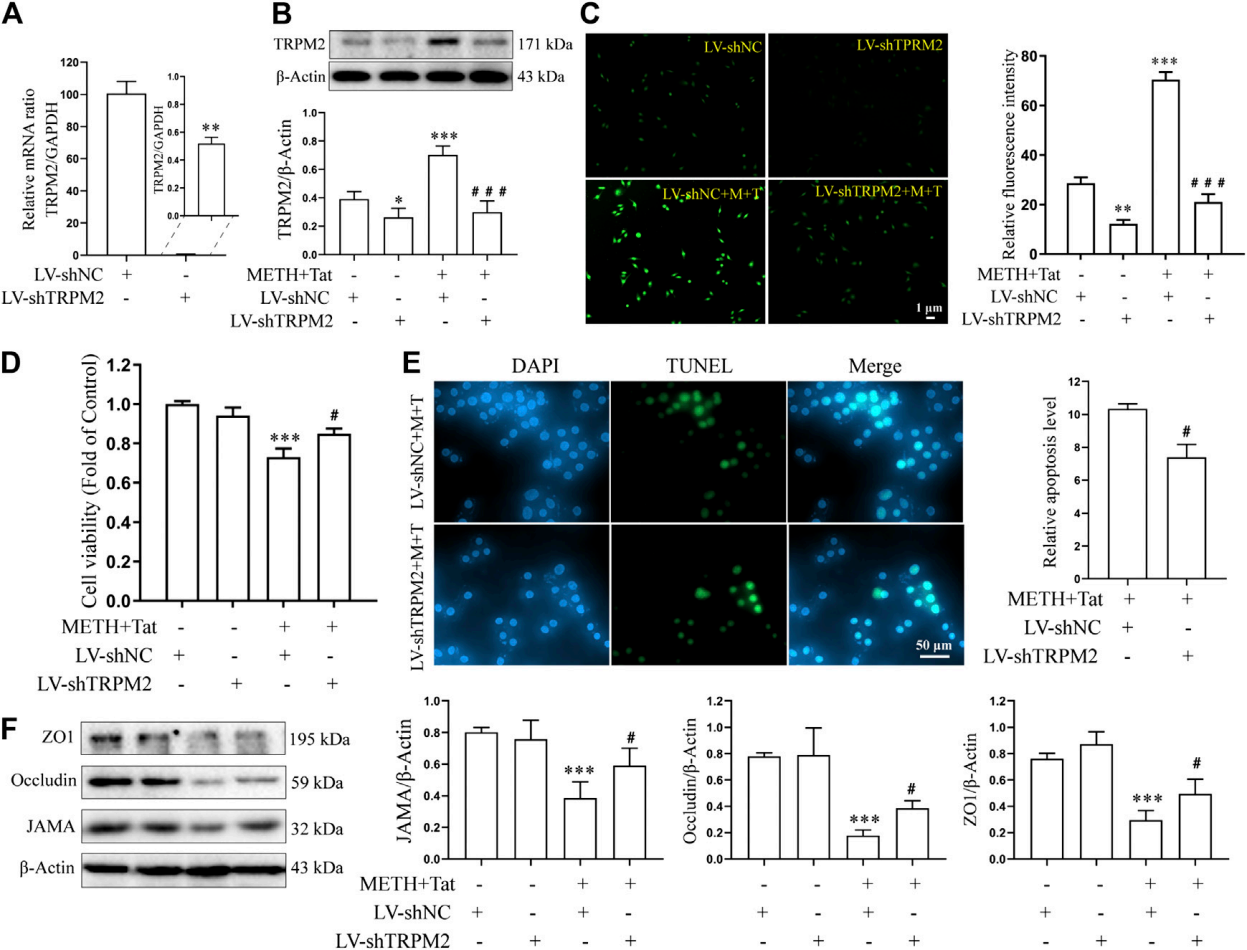
TRPM2 gene knockdown attenuates damage to the BBB synergistically induced by METH and HIV-Tat *in vitro*. The hCMEC/D3 cells were transfected with LV-shTRPM2 or LV-shNC for 48 h before 0.5 mM of METH and 100 nM of HIV-Tat for 24 h. **(A)** Real-time qPCR was performed to determine the TRPM2 mRNA expression level; **(B)** western blot was performed to determine the TRPM2 expression level; **(C)** Ca^2+^ fluorescence was observed via an inverted fluorescence microscope (Scale bar: 1 µm), and fluorescence intensity was analyzed using Image J software; **(D)** cell viability was measured using the CCK8 kit; **(E)** apoptosis was assessed via TUNEL assay (Scale bar: 50 µm), apoptosis level was determined by the number of TUNEL-positive cells expressed as a percentage of the total cell number (analyzed by Image J software); **(F)** Western blot was performed to determine JAMA, Occludin, and ZO1 expression levels. Compared with the LV-shNC, *p < 0.05, **p < 0.01, ***p < 0.001; compared with the LV-shNC + METH + HIV-Tat, ^#^ *p* < 0.05, ^###^ *p* < 0.001, n = 3. LV-shNC = empty vector, LV-shTRPM2 = shTRPM2 lentivirus, LV-shNC + M+T = empty vector+0.5 mM of METH+100 nM of HIV-Tat, and LV-shTRPM2+M+T = shTRPM2 lentivirus+0.5 mM of METH+100 nM of HIV-Tat. The Ca^2+^ fluorescence shown in **(C)** was detected after the cells were transfected with LV-shTRPM2 or LV-shNC for 48 h before 0.5 mM of METH and 100 nM of HIV-Tat for 0.5 h.

Lentivirus transfection technology was next used to investigate whether knockdown TRPM2 gene expression in hCMEC/D3 cells could reduce BBB injury induced by METH and HIV-Tat *in vitro*. Through an inverted fluorescence microscope, the lentivirus was observed to successfully transfect into the cells (Supplementary Figure S1F). qPCR results showed that the TRPM2 mRNA expression level of the LV-shTRPM2 transfected cells was extremely low compared to the negative control group (Figure 6A). After TRPM2 gene expression was knocked down, the METH- and HIV-Tat-induced high expression of TRPM2 protein (Figure 6B) and the increase in intracellular Ca^2+^ fluorescence intensity (METH and HIV-Tat exposure for 0.5 h) (Figure 6C) also decreased significantly, indicating that TRPM2 channels were inhibited. Like the inhibition of the TRPM2 channel via drug intervention, the TRPM2 gene knockdown was observed to improve abnormal cell morphology (Supplementary Figure S1G), viability (Figure 6D), apoptosis levels (Figure 6E), TJ protein expression levels (Figure 6F), and NaF flux (Supplementary Figure S2C) caused by METH and HIV-Tat. These results indicate that the TRPM2 channel regulates METH- and HIV-Tat-induced synergistic injury to the BBB *in vitro*.

### TRPM2 Channel Regulates METH and HIV-Tat Co-Induced Oxidative Stress to Damage the BBB *in vitro*

**FIGURE 7:**
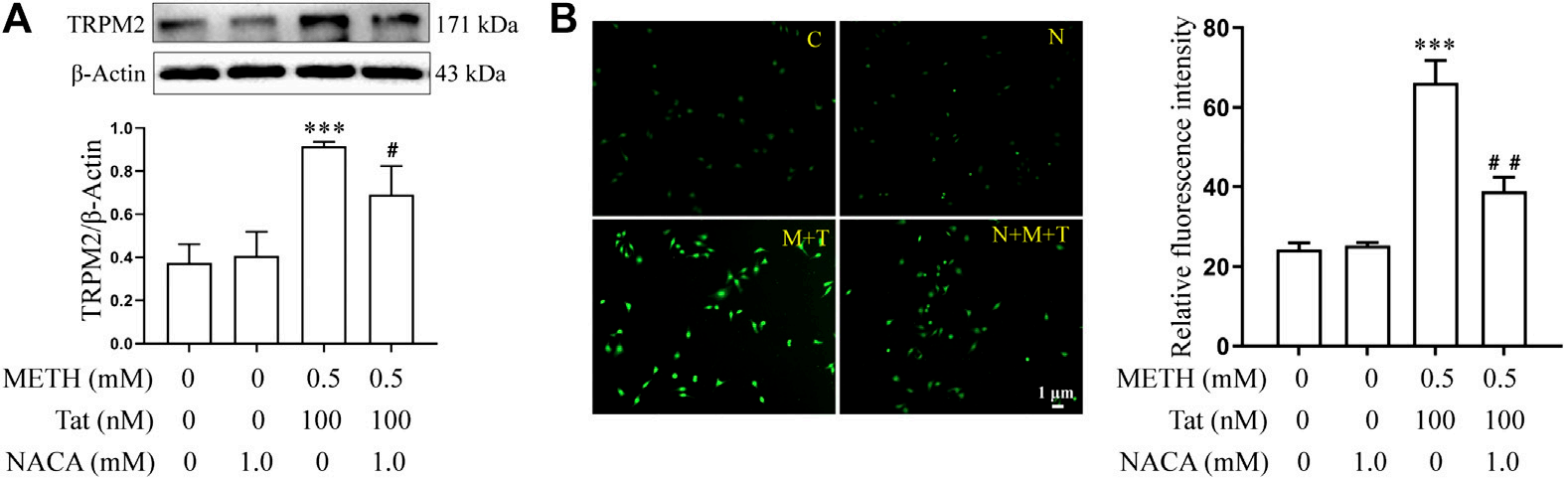
NACA attenuates activation of TRPM2 channel synergistically induced by METH and HIV-Tat. The hCMEC/D3 cells were treated with or without 1.0 mM of NACA for 1 h before 0.5 mM of METH and 100 nM of HIV-Tat for 24 h. **(A)** Western blot was performed to determine the TRPM2 expression level; **(B)** Ca^2+^ fluorescence was observed using an inverted fluorescence microscope (Scale bar: 1 µm), and fluorescence intensity was analyzed using Image J software. Compared with the control, ***p < 0.001; compared with the METH + HIV-Tat, ^#^ *p* < 0.05, ^##^ *p* < 0.01, n = 3. C=Control, N = 1.0 mM of NACA, M + T = 0.5 mM of METH+100 nM of HIV-Tat, and N + M + T = 1.0 mM of NACA + 0.5 mM of METH+100 nM of HIV-Tat. The Ca^2+^ fluorescence shown in **(B)** was detected after the cells were treated with or without 1.0 mM of NACA for 1 h before 0.5 mM of METH and 100 nM of HIV-Tat for 0.5 h.

**FIGURE 8:**
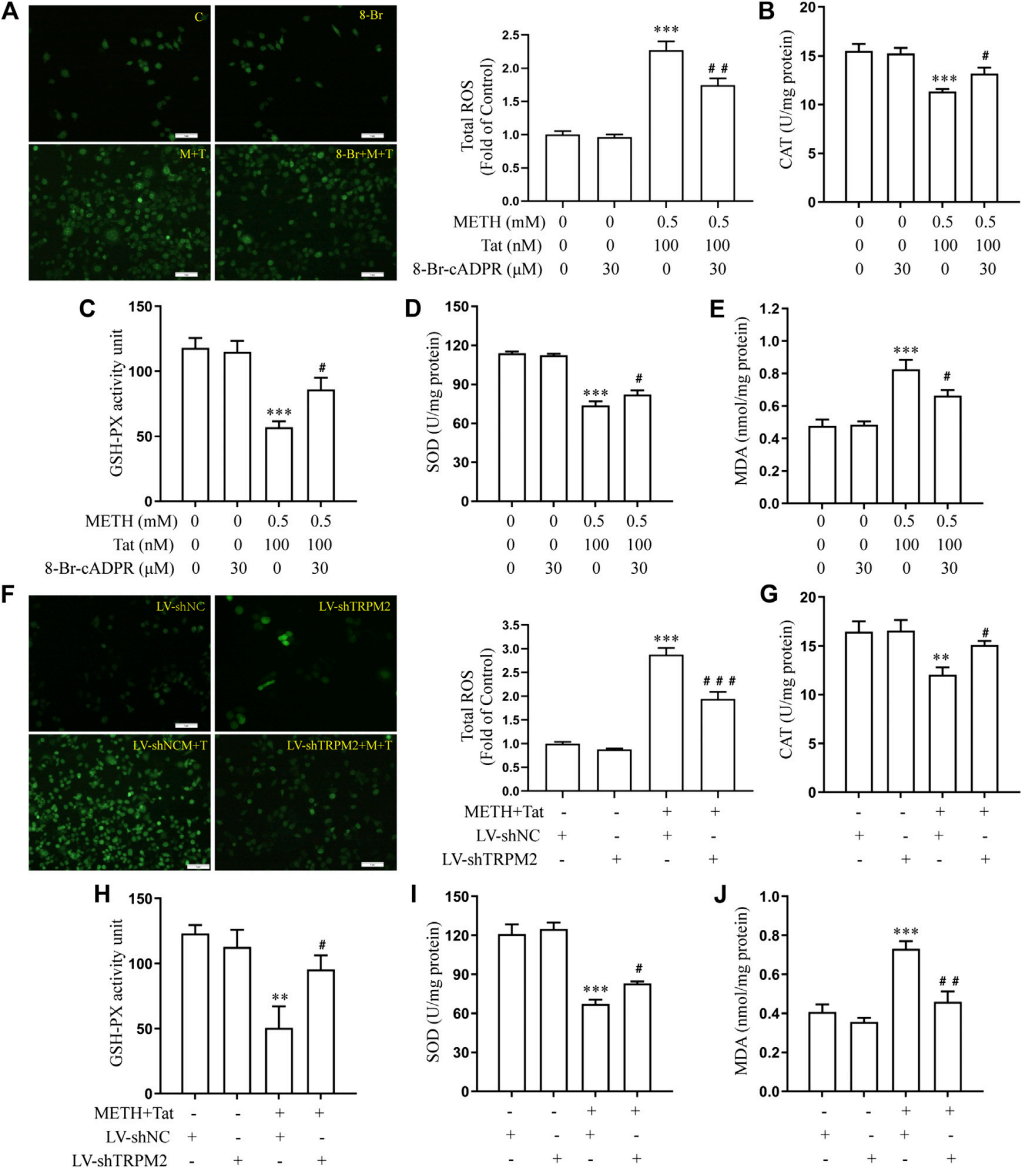
8-Br-cADPR and TRPM2 gene knockdown attenuate oxidative stress responses synergistically induced by METH and HIV-Tat. The hCMEC/D3 cells were transfected with LV-shTRPM2 or LV-shNC for 48 h or treated with or without 30 μM of 8-Br-cADPR for 0.5 h before 0.5 mM of METH and 100 nM of HIV-Tat for 24 h. **(A, F)** ROS fluorescence was observed via an inverted fluorescence microscope (Scale bar: 1 µm), and fluorescence intensity was analyzed using Image J software; **(B–E, G–J)** CAT, GSH-PX, and SOD activity and the level of MDA were measured using commercial kits. Compared with the control or LV-shNC, **p < 0.01, ***p < 0.001; compared with the METH + HIV-Tat or LV-shNC + METH + HIV-Tat, ^#^ *p* < 0.05, ^##^ *p* < 0.01, n = 3. C=Control, 8-Br = 30 μM of 8-Br-cADPR, M + T = 0.5 mM of METH+100 nM of HIV-Tat, and 8-Br + M + T = 30 μM of 8-Br-cADPR+0.5 mM of METH+100 nM of HIV-Tat. LV-shNC = empty vector, LV-shTRPM2 = shTRPM2 lentivirus, LV-shNC + M+T = empty vector+0.5 mM of METH+100 nM of HIV-Tat, and LV-shTRPM2+M+T = shTRPM2 lentivirus+0.5 mM of METH+100 nM of HIV-Tat.

The relationship between TRPM2 channels and oxidative stress was then examined in an *in vitro* model of METH- and HIV-Tat-induced synergistic injury to the BBB. After NACA intervention, the high expression of TRPM2 protein (Figure 7A) and the increase in intracellular Ca^2+^ fluorescence intensity (METH and HIV-Tat exposure for 0.5 h) (Figure 7B) in hCMEC/D3 cells caused by METH and HIV-Tat decreased. On the contrary, oxidative stress also decreased when the TRPM2 channels were suppressed using drugs or knockdown techniques (Figures 8A–J). These results suggest that the TRPM2 channel is not only an effector of oxidative stress, but the channel can in turn affect oxidative stress. In sum, the TRPM2 channel regulates METH and HIV-Tat co-induced oxidative stress to damage the BBB *in vitro*.

### TRPM2 Channel Regulates METH- and HIV-Tat-Induced Synergistic Injury to the BBB *in vivo*

**FIGURE 9:**
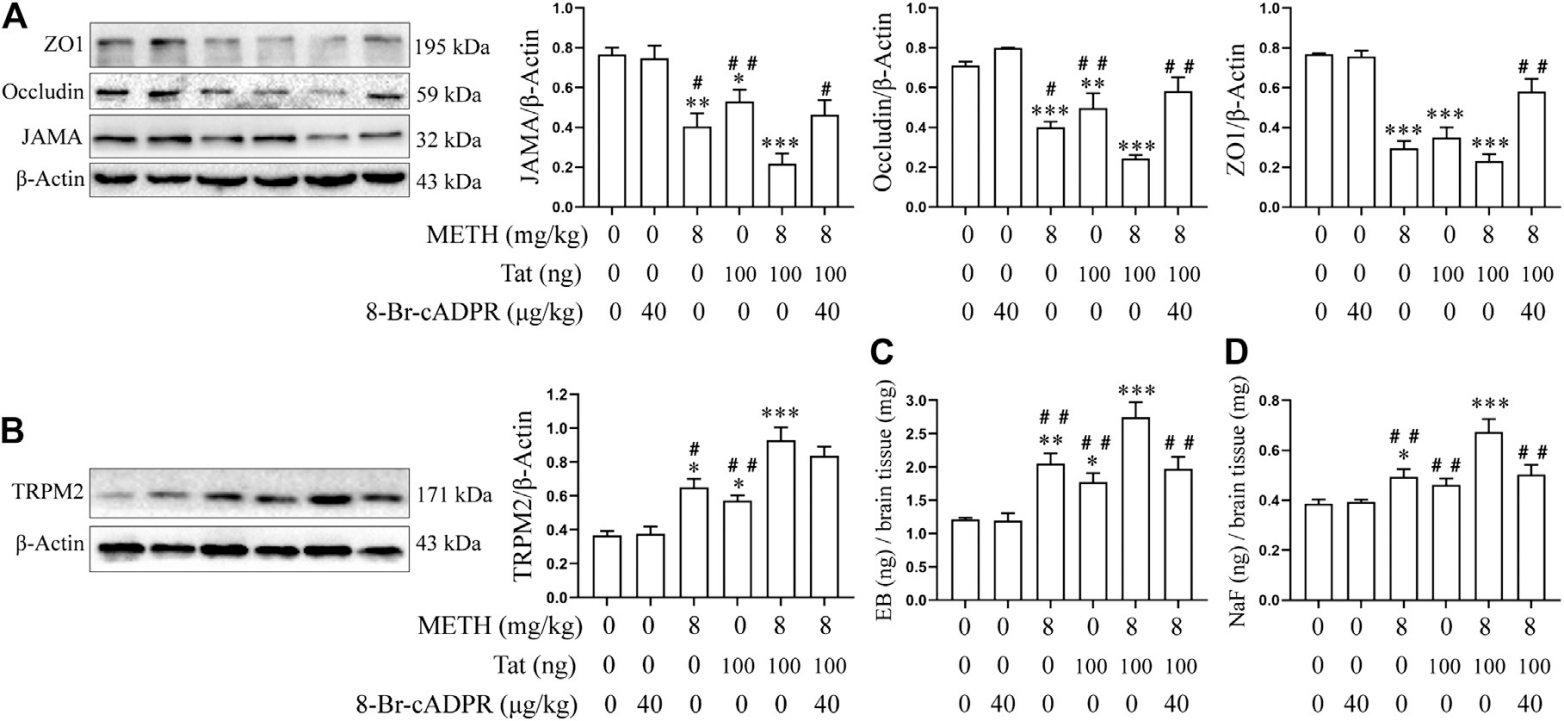
8-Br-cADPR attenuates synergistic METH- and HIV-Tat-induced BBB damage in tree shrews. All the animals were treated with METH and/or HIV-Tat with or without the intervention of 8-Br-cADPR for 10 consecutive days by the above administration regimen, then euthanized 24 h after the last treatment. Six tree shrews in each group were treated with EB or NaF by tail intravenous injection 1 h before the end of the last treatment. **(A,B)** Western blot was performed to determine JAMA, Occludin, ZO1, and TRPM2 expression levels in the prefrontal cortex of the tree shrews; **(C)** EB and **(D)** NaF quantitative analyses in a whole-brain context were used to detect the permeability of BBB. Compared with the control, *p < 0.05, **p < 0.01, ***p < 0.001; compared with the METH + HIV-Tat, ^#^ *p* < 0.05, ^##^ *p* < 0.01, n = 3.

The tree shrews were administered drugs according to the experimental procedure described above to determine whether METH and Tat co-damage the BBB, as well as to explore the role of the TRPM2 channel in this process. Compared with group C, the JAMA, Occludin, and ZO1 expression levels in the prefrontal cortex of the tree shrews in groups M, T, and M + T decreased significantly (Figure 9A), while the EB and NaF content in the shrews’ brain tissues increased significantly (Figures 9C,D). These changes were most prominent in the tree shrews from group M + T, where the effect values were 0.804 (JAMA), 0.817 (Occludin), 0.973 (ZO1), 0.707 (EB), and 0.533 (NaF), indicating a synergistic effect (Figures 9A,C,D). This suggests that METH and HIV-Tat can co-damage the tree shrew’s BBB. We also found that Both METH and HIV-Tat induced high TRPM2 protein expression in the prefrontal cortex of the tree shrews (Figure 9B). This effect was most prominent in the group M + T, indicating a synergistic effect (the effect value was 0.712) (Figure 9B). TJ protein expression, meanwhile, increased significantly after the advance intervention of 8-BR-CADPR (Figure 9A). EB and NaF content also fell significantly after the intervention (Figures 9C,D), suggesting that BBB injury had improved. These results suggest that the TRPM2 channel can regulate METH- and HIV-Tat-induced synergistic injury to the BBB in tree shrews.

## Discussion

METH abuse and HIV infection are two major public health problems in the world today, and these issues are magnified by the high proportion of METH abusers within the HIV-positive population. METH abuse is closely related to increased HIV replication, enhanced HIV-Tat mediated neurotoxic effects, and neurocognitive impairment (Mediouni et al., 2015). A normally functioning BBB with a complete structure can maintain the internal and external environment stability of the CNS and protect it from external stimulation. METH and HIV-Tat, however, damage the BBB and greatly increase the incidence of HAND (Li et al., 2018b) (though the specific mechanism for this process has not been clarified in the existing literature). This study reveals that METH and HIV-Tat can co-induce oxidative stress to damage the BBB *in vitro*; specifically, the abnormal morphology of the hCMEC/D3 cells, decreased cell viability, increased apoptosis levels, reduced TJ protein expression levels, and increased the flux of NaF across the hCMEC/D3 cells. Drug interventions and gene knockdowns were shown to inhibit the TRPM2 channel, reducing injury to the BBB *in vitro*. *In vivo* experiments also proved that METH and HIV-Tat can increase BBB permeability and down-regulate the TJ protein expression levels in the tree shrews. Inhibiting the TRPM2 channel with drugs was shown to reduce BBB damage induced by METH and HIV-Tat in tree shrews.

As BMECs are an important part of the BBB, many studies have used primary BMEC (Avraham et al., 2004; Xu et al., 2012; Ma et al., 2014; Jiang et al., 2017b) or BMEC cell lines (Fernandes et al., 2014; Patel et al., 2017) to establish *in vitro* models of METH- or HIV-Tat-induced injury to the BBB. Qie et al. (Qie et al., 2017) found that bEnd.3 cells treated with 1.0 mM of METH for 24 h increased apoptosis and decreased viability, TEER, and TJ protein expression. Fernandes et al. (Fernandes et al., 2014) found that bEnd.3 cells lost and redistributed Claudin5 after exposure to 0.5 mM of METH for 24 h. Another study used the primary rats BMEC. Here, 0.1 mM of METH increased apoptosis and permeability while reducing TEER for 24 h (Jumnongprakhon et al., 2016). Besides, HIV-Tat increased apoptosis, decreased cell viability, and down-regulated Occludin expression (both mRNA and protein) of human BMECs (Xu et al., 2012; Ma et al., 2014).

Few studies, however, have reported the effects of both METH and HIV-Tat on BBB models *in vitro*. In the present research, hCMEC/D3 cells—which originate from the human microvascular endothelium, are similar to the BMECs in the BBB, and can express various TJ proteins (Weksler et al., 2013)—were studied. The cells were treated with gradient concentrations of METH (0.05 to 2.0 mM) or HIV-Tat (25 to 200 nM) for 24 h, and the expression levels of JAMA, Occludin, and ZO1 decreased in a dose-dependent manner. Compared with the control group, the cells treated with 0.5 mM of METH or 100 nM of HIV-Tat 24 h exhibited significantly reduced TJ protein expression levels. After combining the 0.5 mM of METH and 100 nM of HIV-Tat treat the cells for 24 h, the abnormal cell morphology, apoptosis levels, TJ protein expression levels, and NaF flux were more obvious than when METH or HIV-Tat were applied alone. This indicated that when combined, they caused more serious effects (effect value < 1), prompting the hypothesis that METH and HIV-Tat can co-induce BBB damage *in vitro*.

At present, not much research studies the link between synergistic BBB injury and METH and HIV-Tat through animal experiments. In one previous study, SD rats were given 10 mg/kg of METH and/or 50 ng/kg of HIV-Tat daily. After continuous treatment for seven days, BMEC edemas in the rats’ cortical areas were observed via transmission electron microscopy, accompanied by several drinking vesicles. BBB permeability increased significantly, while the expression levels of Occludin, JAMA, claudin-5, and ZO1 decreased significantly. These changes were most serious when METH and HIV-Tat were combined (Li et al., 2018b). In the present study, the tree shrew was used as an experimental animal to further verify whether METH and HIV-Tat can synergistically damage the BBB. Like previous reports, we found that METH and/or HIV-Tat administration for 10 consecutive days led to significant decreases in the expression levels of JAMA, Occludin, and ZO1 in the tree shrews’ prefrontal cortex and increases in the concentrations of EB and NaF in the shrews’ brain tissues. These changes are more serious when METH and HIV-Tat were used together (all effect values <1), indicating that METH and HIV-Tat can co-damage the BBB in the tree shrews.

The oxidative stress response is one of the mechanisms of METH and HIV-Tat that cause neurotoxicity (Mediouni et al., 2015; Yang et al., 2018). Both METH and HIV-Tat can induce excessive ROS production, impairing the defense abilities of antioxidant enzymes such as GSH-PX and SOD, then inducing autophagy and apoptosis of nerve cells by inhibiting the mTOR (Zeng et al., 2018). NADPH oxidase 2(NOX2) activation can cause intracellular ROS accumulation. This further inhibits the nuclear factor erythroid-2-related factor 2(Nrf2) from entering the cell nucleus, leading to the decrease of heme oxygenase-1(HO-1), NAD(P)H quinone oxidoreducpase-1 (NQO-1), γ-glutamylcysteine synthetase (γ-GCS), and SOD. This pathway is responsible for METH-induced oxidative stress injury in primary rats BMEC (Jumnongprakhon et al., 2016). The oxidative stress response induced by HIV-Tat is also related to damaged Nrf2 balances, manifests in the accumulation of ROS and nitric oxide in nerve cells, decreased GSH levels, and increased GSSG levels (Kim et al., 2015). Besides, Flora et al. (Flora et al., 2003) confirmed that METH and/or HIV-Tat can induce oxidative stress and activate redox-regulated transcription factors and inflammatory genes in the mouse brain.

The role of oxidative stress in inducing BBB injury has been widely recognized (Pun et al., 2009; Northrop and Yamamoto, 2015; Namyen et al., 2020). Toborek et al. (Toborek et al., 2003) suggested that the increase of cellular oxidative stress may be a mechanism of BBB injury induced by HIV-Tat. A previous study confirmed that METH and HIV-Tat can co-damage the BBB in SD rats. NACA not only corrected the oxidative stress injury but also alleviated damage to the BBB induced by METH + HIV-Tat + GP120 (Banerjee et al., 2010). In the present research, similar results were obtained at a cellular level. We found that METH and HIV-Tat could both induce decreased CAT, GSH-PX, and SOD activity in hCMEC/D3 cells, as well as elevated ROS and MDA levels. These changes were most obvious (effect value <1) when METH and HIV-Tat acted together, suggesting that the combination synergistically induces the oxidative stress reaction. Here, NACA intervention not only reduced the severity of oxidative stress but also the damage to the BBB. These mediating effects were observed in the cells’ improved morphology, decreased apoptosis levels, increased cell viability, TJ protein expression levels, and decreased the flux of NaF across the hCMEC/D3 cells.

As oxidative stress reaction cell receptors, TRPM2 channels can mediate the oxidative stress reaction—which in turn can mediate the occurrence and development of some nervous system diseases. Ostapchenko et al. (Ostapchenko et al., 2015) found that the landmark toxic protein present in Alzheimer's disease (AD), the amyloid β-protein (Aβ), can promote TRPM2 channel activation. In AD mice models, genetic ablation of TRPM2 was found to lighten the protein’s neurotoxic effects and improve age-related memory defects. Intracellular ROS aggregation can activate NLRP3 inflammasomes by stimulating TRPM2 channels (Wang et al., 2020), or activate microglia cells and induce TNF- production (Alawieyah Syed Mortadza et al., 2018), a neuroinflammatory response induced by Aβ that appears to exacerbate AD (Aminzadeh et al., 2018). With Parkinson’s disease (PD), there are also abnormal expressions and functions of the TRPM2 channel (Hermosura et al., 2008). Another mouse model of hypoxia and ischemic brain injury observed that the cerebral infarction area of TRM2 ^+/-^ and TRM2 ^-/-^ became reduced, the expression of inflammatory markers decreased, and sensorimotor functions improved (Huang et al., 2017). These studies all indicate that the TRPM2 channel is a potential drug intervention target for the treatment of a variety of neurodegenerative diseases (Wang et al., 2016; Yamamoto and Shimizu, 2016; Belrose and Jackson, 2018; Sita et al., 2018).

Research has shown that activating TRPM2 channels can also mediate H_2_O_2_-induced increased permeability of pulmonary artery endothelial cells and increased apoptosis (Hecquet et al., 2008; Hecquet and Malik, 2009; Hecquet et al., 2014). The TRPM2-activated Ca^2+^ signaling and VE-cadherin phosphorylation can regulate endotoxin-induced transmigration of polymorphonuclear neutrophils, resulting in the disassembly of adherens junctions and opening of the paracellular pathways in human lung microvascular endothelial cells (Mittal et al., 2017). Oxidative stress can also induce nitration of the TRPM2 channel Y1485 tyrosine, which plays an important role in peripheral cell damage in the BBB (Jiang et al., 2017a). The present study reveals that treatments using a gradient concentration of METH (0.05 to 2.0 mM) or HIV-Tat (25 to 200 nM) for 24 h increases the expression level of TRPM2 in hCMEC/D3 cells. The combination of 0.5 mM METH and 100 nM HIV-Tat increases the expression level of TRPM2 protein significantly (effect value <1). Also, both 0.5 mM of METH and/or 100 nM of HIV-Tat induce intracellular Ca^2+^ concentration to increase when treated for 0.5 h. When combined, METH and HIV-Tat had a synergic effect (effect value <1), suggesting that TRPM2 channels can be activated by METH, HIV-Tat, or both. Interventions using 8-BR-ADPR or TRPM2 gene expression knockdown to inhibit the TRPM2 channel improved the abnormal cell morphology caused by METH and HIV-Tat. The interventions also improved the observed decreased cell viability, increased apoptosis levels, decreased TJ protein expression levels, and increased NaF flux. *In vivo* experiments revealed that both METH and/or HIV-Tat can induce high expressions of TRPM2 proteins in the prefrontal cortex of tree shrews, with a synergistic effect (effect value <1). Following drug administration to inhibit the TRPM2 channels, METH- and HIV-Tat-induced BBB injury improved, as shown by increased JAMA, Occludin, and ZO1 expression levels and decreased EB and NaF concentrations. This confirmed that the TRPM2 channel can regulate METH and HIV-Tat co-damage to the BBB both *in vivo* and *in vitro*.

The relationship between the oxidative stress response and TRPM2 channels when METH and HIV-Tat co-damage the BBB was also examined via an *in vitro* model. NACA intervention reduced TRPM2 protein expression and TRPM2 channel activation caused by M + T. Intervention with 8-BR-ADPR or TRPM2 gene expression knockdown to inhibit the TRPM2 channel also attenuated oxidative stress injury caused by M + T. Previous studies had similar results in finding that H_2_O_2_ can activate protein kinase C (PKC) and NOX to produce excessive ROS, thus activating the TRPM2 channel. The TRPM2 channel can mediate lysosomal dysfunction and release lysosomal Zn^2+^, but also induce mitochondrial Zn^2+^ accumulation, triggering mitochondrial dysfunction, and ROS production. Mitochondrial dysfunction can also enhance NOX-mediated ROS production and the oxidative stress response, which induces the delayed death of SH-SY5Y cells (Li and Jiang, 2019). A similar positive feedback mechanism was observed when Zn^2+^ induced microglia death by TRPM2 channel activation (Mortadza et al., 2017). Ozkaya et al. (Ozkaya and Naziroglu, 2020) found that oxidative stress-dependent TRPM2 activation plays an important role in cisplatin-induced optic neuron death in mice. Inhibition of the TRPM2 channel improved mitochondrial membrane depolarization caused by cisplatin, reduced ROS levels in the mitochondria and cytoplasm, and reduced optic nerve injury. These results are consistent with the results of the present study, suggesting that TRPM2 channels are not only effectors of oxidative stress response but also interact with each other.

Claudin-5 is a tight junction protein regulating the integrity and permeability of BBB. It warrants further investigation to characterize the role of claudin-5 on BBB injury by METH and HIV-Tat. Previous studies indicated that pro-inflammatory stimulation can down-regulate TJ protein expression levels in hCMEC/D3 cells (Lopez-Ramirez et al., 2012). Aβ peptides can increase hCMEC/D3 monolayer permeability (Tai et al., 2010). There are reports to study the effects of Tat and METH on BBB integrity and function using hCMEC/D3 cells (Patel et al., 2017). However, it is of biological significance to verify the effects of HIV-Tat and METH on BBB using primary human brain endothelial cells. Additionally, it warrants further investigation into the biological relevance and the characterization of the hCMEC/D3 for the BBB study. Also, the use of simple DMEM/high glucose medium with 10% FBS with human basic fibroblast growth factor promotes cell growth, but it lacks basic supplements needed for the culture of this cell type which might interfere with basic BBB properties. In the future, we plan to characterize the model with the suggested and published medium components (Weksler et al., 2005).

In sum, this study confirms that METH and HIV-Tat can co-induce oxidative stress to damage the BBB; the TRPM2 channel can regulate this BBB injury process. This research provides a new theory for explaining the mechanism of synergistic BBB injury by METH and HIV-Tat, and it presents the TRPM2 channel as a promising drug intervention target to reduce BBB injury and neuropsychiatric symptoms in HIV-infected METH abusers. Future research into synergistic BBB injury induced by METH and HIV-Tat proteins could further explore the specific interaction mechanism between the oxidative stress response and the TRPM2 channel, as well as the exact mechanism of the TRPM2 channel in mediating BBB injury.

## Data Availability

The original contributions presented in the study are included in the article/Supplementary Material, further inquiries can be directed to the corresponding authors.
