# Supplementary figures and images for: Methamphetamine and HIV-Tat Protein Synergistically Induce Oxidative Stress and Blood-Brain Barrier Damage via Transient Receptor Potential Melastatin 2 Channel
Source: Front Pharmacol. 2021 Mar 17;12:619436. doi: 10.3389/fphar.2021.619436 (PMC8010131; doi:10.3389/fphar.2021.619436)

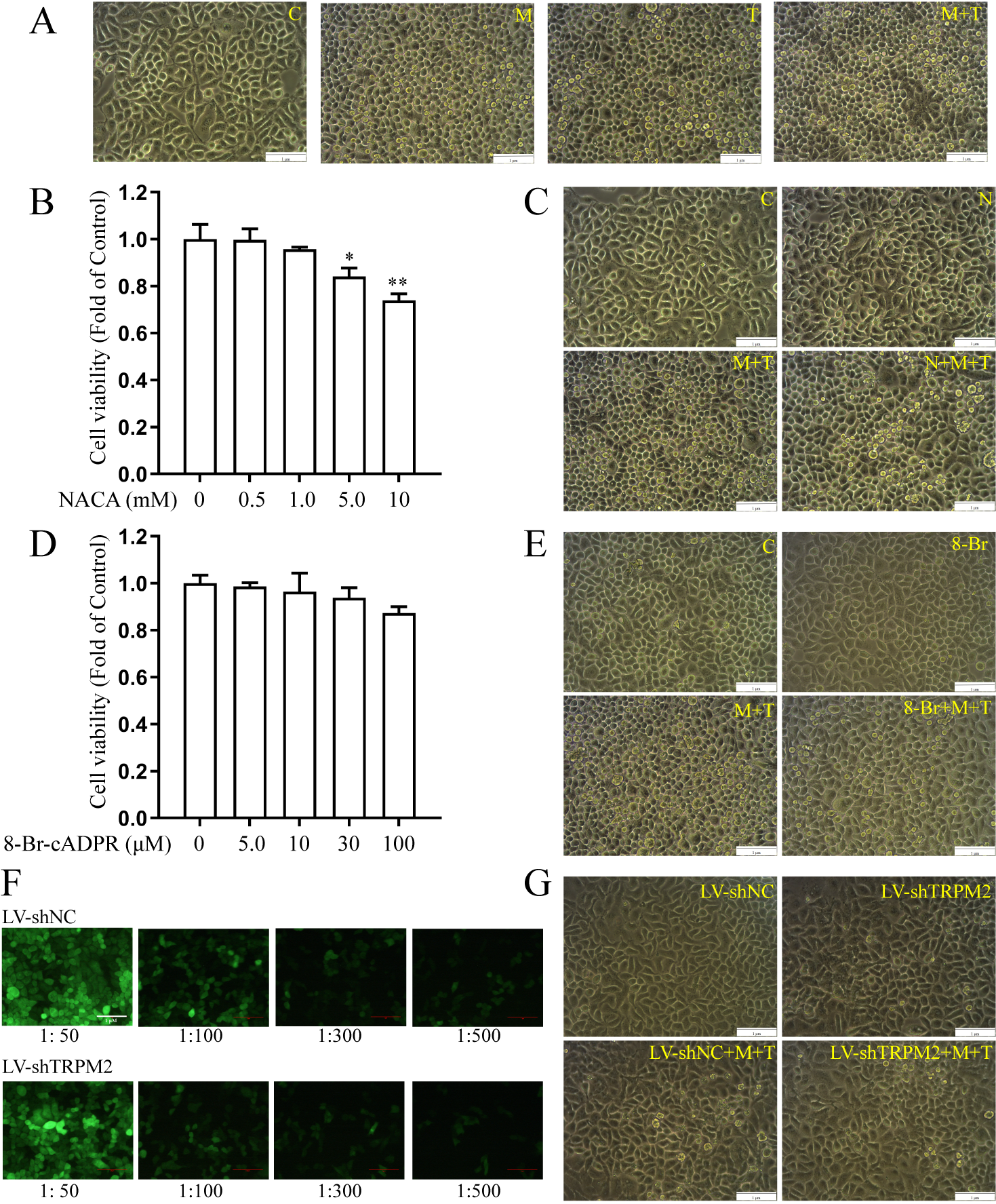

Supplement: Supplementary file 1 [file image1.tif]

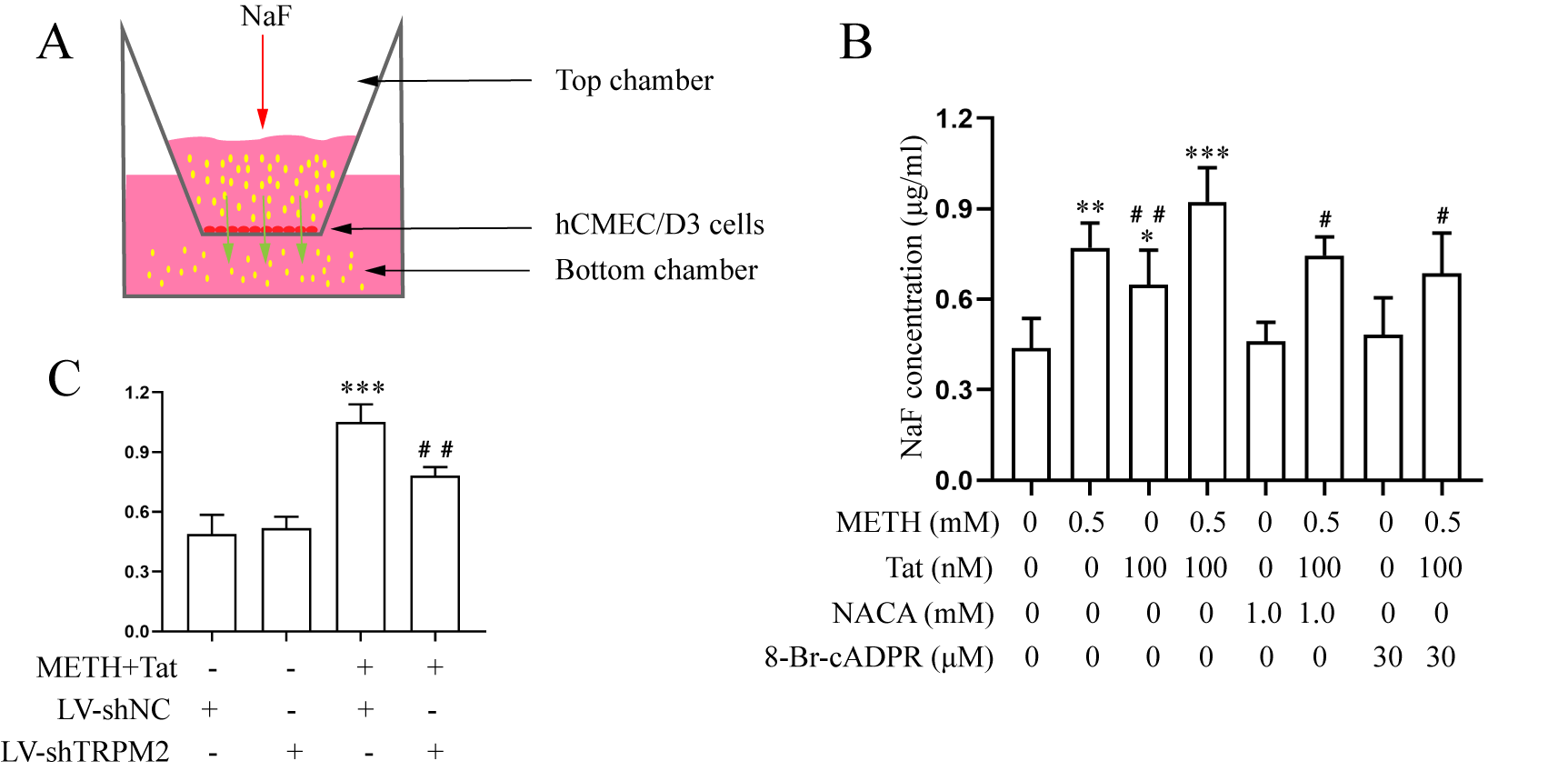

Supplement: Supplementary file 2 [file image2.tif]

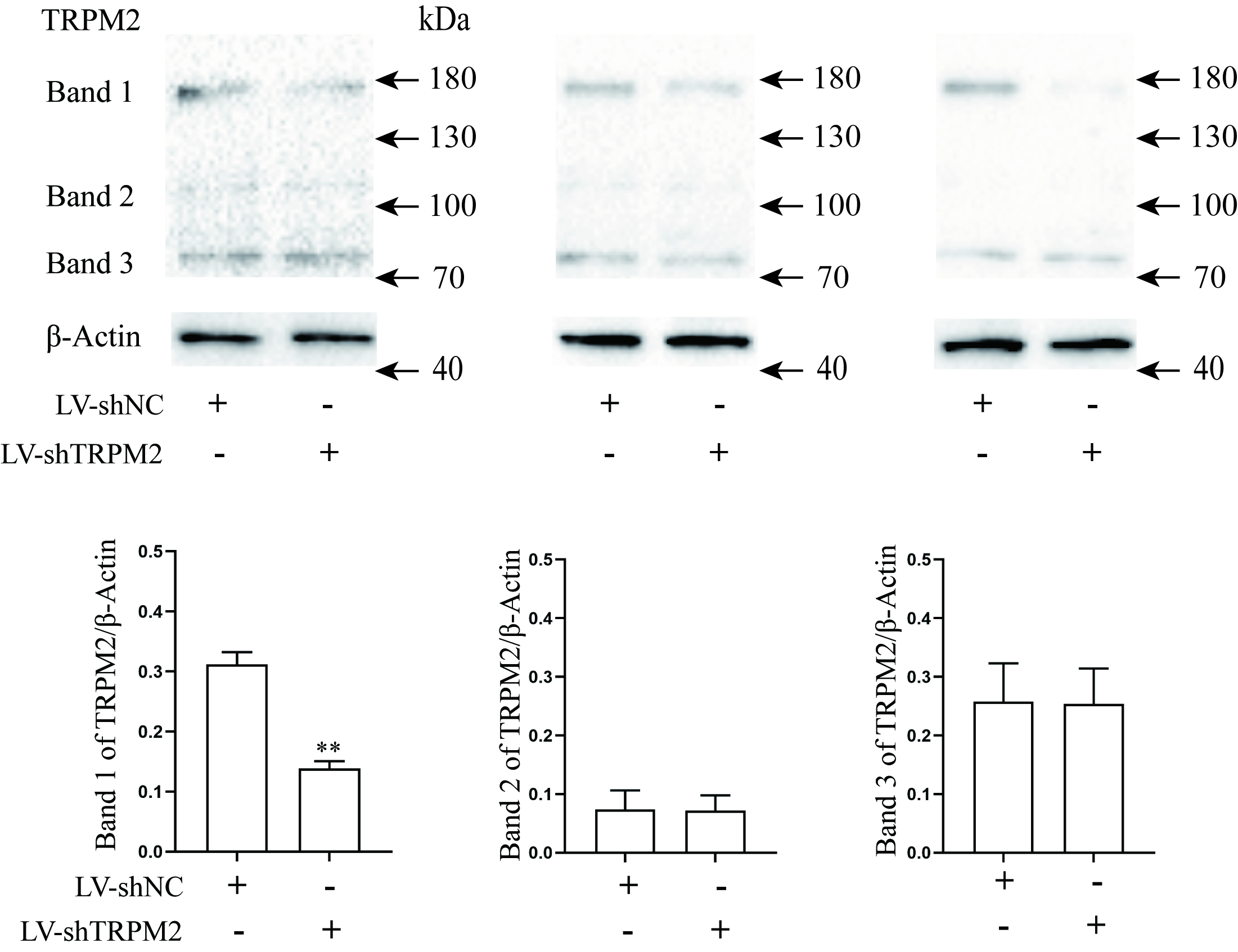

Supplement: Supplementary file 3 [file image3.tif]
